# 3K3A-Activated Protein C Protects the Blood-Brain Barrier and Neurons From Accelerated Ischemic Injury Caused by Pericyte Deficiency in Mice

**DOI:** 10.3389/fnins.2022.841916

**Published:** 2022-03-30

**Authors:** Yaoming Wang, Kassandra Kisler, Angeliki Maria Nikolakopoulou, Jose A. Fernandez, John H. Griffin, Berislav V. Zlokovic

**Affiliations:** ^1^Department of Physiology and Neuroscience, Keck School of Medicine, Zilkha Neurogenetic Institute, University of Southern California, Los Angeles, CA, United States; ^2^Department of Molecular Medicine, The Scripps Research Institute, La Jolla, CA, United States; ^3^Division of Hematology/Oncology, Department of Medicine, University of California, San Diego, San Diego, CA, United States

**Keywords:** pericyte, ischemic stroke, blood-brain barrier, neurons, activated protein C

## Abstract

Pericytes, mural cells of brain capillaries, maintain the blood-brain barrier (BBB), regulate cerebral blood flow (CBF), and protect neurons against ischemic damage. To further investigate the role of pericytes in ischemia, we induced stroke by 45-min transient middle cerebral artery occlusion (tMCAo) in 6-month-old pericyte-deficient *Pdgfrb*^ + ⁣/−^ mice and control *Pdgfrb^+/+^* littermates. Compared to controls, *Pdgfrb*^ + ⁣/−^ mice showed a 26% greater loss of CBF during early reperfusion, and 40–50% increase in the infarct and edema volumes and motor neurological score 24 h after tMCAo. These changes were accompanied by 50% increase in both immunoglobulin G and fibrinogen pericapillary deposits in the ischemic cortex 8 h after tMCAo indicating an accelerated BBB breakdown, and 35 and 55% greater losses of pericyte coverage and number of degenerating neurons 24 h after tMCAo, respectively. Treatment of *Pdgfrb*^ + ⁣/−^ mice with 3K3A-activated protein C (APC), a cell-signaling analog of plasma protease APC, administered intravenously 10 min and 4 h after tMCAo normalized CBF during the early reperfusion phase and reduced infarct and edema volume and motor neurological score by 55–60%, with similar reductions in BBB breakdown and number of degenerating neurons. Our data suggest that pericyte deficiency results in greater brain injury, BBB breakdown, and neuronal degeneration in stroked mice and that 3K3A-APC protects the brain from accelerated injury caused by pericyte deficiency. These findings may have implications for treatment of ischemic brain injury in neurological conditions associated with pericyte loss such as those seen during normal aging and in neurodegenerative disorders such as Alzheimer’s disease.

## Introduction

Pericytes, perivascular mural cells that grow along and enwrap brain capillary vessels, are essential to maintenance of blood-brain barrier (BBB) integrity (Armulik et al., 2010; Bell et al., 2010; Daneman et al., 2010; Nikolakopoulou et al., 2019). Pericytes also play an important role in cerebral blood flow (CBF) regulation, where the cells are thought to modulate capillary vessel tone or diameter, thereby influencing CBF through the capillary bed (Hall et al., 2014; Kisler et al., 2017a,b, 2020; Sweeney et al., 2018; Nelson et al., 2020; Hartmann et al., 2021). Pericytes have been implicated in the “no-reflow” phenomenon after recanalization of occluded arteries, where capillary constrictions due to ischemia-induced pericyte contraction remain after reperfusion (Yemisci et al., 2009), and pericytes may die in rigor in the contracted state (Hall et al., 2014), disrupting CBF reperfusion and further contributing to the ischemic injury.

Loss of brain capillary pericyte coverage or cell numbers has been observed following stroke in humans and mice (Fernández-Klett et al., 2013; Nirwane et al., 2019; Ding et al., 2020; Zhang et al., 2020; Tsao et al., 2021) and after chronic hypoperfusion in mice (Liu et al., 2019). BBB disruption and/or capillary loss frequently accompanied pericyte loss in these conditions (Fernández-Klett et al., 2013; Nirwane et al., 2019; Ding et al., 2020; Zhang et al., 2020; Tsao et al., 2021). Pericytes or cells expressing pericyte markers also contribute to the subacute and chronic glial scarring phases of stroke recovery (Göritz et al., 2011; Fernández-Klett et al., 2013; Cheng et al., 2018). Together these data indicate that pericytes are sensitive to ischemic conditions and can play different dynamic roles in stroke pathology.

Platelet-derived growth factor receptor β (PDGFRβ) is highly expressed in pericytes, and mice lacking one copy of the *Pdgfrb* gene encoding PDGFRβ develop chronic pericyte loss and BBB breakdown (Bell et al., 2010; Kisler et al., 2017b). Pericyte loss has been reported in several neurological disorders including Alzheimer’s disease (AD), amyotrophic lateral sclerosis (ALS), and other neurodegenerative disorders as recently reviewed (Sweeney et al., 2019). Additionally, shedding of soluble PDGFRβ is associated with increased BBB leakage and cognitive decline in humans (Montagne et al., 2015, 2020; Sagare et al., 2015; Nation et al., 2019). However, the extent to which pericyte loss may play a role in the pathogenesis of ischemic stroke still warrants further investigations.

Here, we use *Pdgfrb*^ + ⁣/−^ mice with chronic pericyte deficiency to investigate the effect of pericyte loss on stroke outcome. We also study whether recombinant murine 3K3A-activated protein C (APC), a signaling-selective APC analog (Mosnier et al., 2007, 2014; Guo et al., 2013) with diminished (<25%) anticoagulant activity but normal cell signaling cytoprotective activities (Mosnier et al., 2004), can protect *Pdgfrb*^ + ⁣/−^ mice from ischemic injury. Previous studies indicated that 3K3A-APC is protective in rodent models of large artery infarcts (Guo et al., 2009a; Wang et al., 2012, 2013; Sinha et al., 2018), white matter stroke (Huuskonen et al., 2022), traumatic brain injury (Walker et al., 2010), ALS (Zhong, 2009; Shi et al., 2019), and AD (Lazic et al., 2019). 3K3A-APC is safe and well-tolerated and has an established safety and pharmacokinetic profile in humans (Williams et al., 2012; Lyden et al., 2013), and it reduces intracerebral bleeding in ischemic stroke patients (Lyden et al., 2019), consistent with its BBB protection in stroke models (Wang et al., 2013; Amar et al., 2018; Griffin et al., 2018).

## Materials and Methods

### Animals

All procedures have been approved by the Institutional Animal Care and Use Committee at the University of Southern California per the National Institutes of Health guidelines. Mice were housed in plastic cages on a 12-h light cycle, with *ad libitum* access to food and water. Six-month-old *Pdgfrb* heterozygous knockout (*Pdgfrb*^ + ⁣/−^) mice and littermate controls (*Pdgfrb*^+/+^) were used in the study. Animals were randomized for treatment groups. All experiments were blinded with respect to the operators responsible for surgical procedures and outcome assessments.

### 3K3A-Activated Protein C

Murine recombinant 3K3A-APC (KKK192-194AAA) was prepared in the Griffin laboratory as described previously (Mosnier et al., 2004).

### Proximal Transient Middle Cerebral Artery Occlusion

Mice were anesthetized intraperitoneally with 100 mg ketamine/10 mg xylazine per kg body weight. Rectal temperature was maintained between 36.5 and 37.0°C during the procedure using a feedback-controlled heating system. The MCA was occluded for 45 min using a silicon-coated nylon monofilament (DOCCOL CO.) as described (Wang et al., 2012). CBF was monitored by laser Doppler flowmetry (LDF). See below for details. Only mice with adequate MCAo as evidenced by ≥80% drop in the CBF were included in the study. 3K3A-APC (0.2 mg/kg) or vehicle was administrated intravenously 10 min and 4 h after tMCAo initiation *via* tail vein. For 10-min post-tMCAo injection mice were still under the initial surgical anesthesia and were not re-anesthetized again for the 4-h injection. Mice were euthanized 8 and 24 h after the tMCAo for tissue analysis as detailed below.

### Cerebral Blood Flow Measurements

Cerebral blood flow was measured by LDF (Transonic Systems Inc., Ithaca, NY, United States) using a flexible fiberoptic probe (0.5 mm in diameter). The MCA was visible through the temporal semitranslucent surface of the skull. The laser Doppler probe was affixed to the left side of the temporal skull surface over the origin of the MCA cortical bifurcation. The initial drop in CBF after MCAo was assessed by LDF. LDF changes were measured during 45 min of ischemia and up to 30 min after reperfusion onset. Relative drop in CBF after tMCAo was expressed as a ratio of CBF after occlusion or during reperfusion compared to baseline CBF before occlusion that is arbitrarily taken as 100.

### Motor Neurological Deficit Score

Motor neurological deficit was evaluated 24 h after stroke using the following scale (Wang et al., 2012): no neurological deficit, 0; failure to extend left forepaw fully, 1; turning to left, 2; circling to left, 3; unable to walk spontaneously, 4; and stroke-related death, 5.

### Cresyl Violet Staining and Neuropathological Analysis

Thin 20-μm cryostat sections from 5 equidistant rostrocaudal brain levels (Zeng et al., 2019), at −1.6, −0.8, 0, 0.8, and 1.6 mm from bregma, fixed by methanol and stained with the Cresyl Echt Violet staining kit (American-MasterTech, catalog # AHC0443). Sections were digitized and transformed into gray scale, and the border between infarct and non-infarct tissue was outlined using ImageJ. The injury volume, the infarct volume, and the edema volume were measured on those coronal sections using cresyl-violet staining as previously described (Wang et al., 2012). The injury volume (cubic millimeters) was calculated by multiplying the surfaces of all injured areas in square millimeters by the thickness of brain sections. The infarction volume was obtained by subtracting the edema volume from the injury volume. The edema volume (tissue swelling) was calculated by subtracting the volume of the contralateral non-ischemic hemisphere from the volume of the ipsilateral ischemic hemisphere as previously described (Wang et al., 2009).

### Immunohistochemistry

At 8 and 24 h after tMCAo, mice were anesthetized and transcardially perfused with PBS. Mouse brains were quickly removed, flash-frozen over dry ice, and stored in −80°C until use. Cryostat sections were cut at 20 μm thickness. Mouse brain sections were fixed by methanol and were blocked with 5% normal donkey serum (Vector Laboratories) and 0.1%Triton-X in 0.01M PBS and incubated with primary antibodies diluted in blocking solution overnight at 4°C. Primary antibodies used in this study include rat anti-mouse CD31 (BD Pharmingen, catalog # 550274), goat anti-mouse CD13 (R&D systems, catalog # AF2335), and rabbit anti-human fibrinogen (Dako, catalog # A0080). After three washes with PBS, sections were incubated with the secondary antibodies for 1 h, including Alexa 568-conjugated donkey anti-rabbit (Thermo Fisher Scientific, catalog # A10042), Alexa 647-conjugated donkey anti-goat (Thermo Fisher Scientific, catalog # A-21447), and Alexa 488-conjugated donkey anti-rat (Thermo Fisher Scientific, catalog # A-21208). Alexa 568-conjugated donkey anti-mouse (Thermo Fisher Scientific, catalog # A10037) was used for IgG staining. All images were taken with the Zeiss 510 confocal microscopy or using the BZ 9000 all-in-one Fluorescence Microscope from Keyence (Osaka, Japan), and analyzed using NIH ImageJ software.

### Quantification Analysis

For quantification of extravascular leakages, pericyte coverage, microvascular length, and the number of degenerating neurons (see below), 20 μm thick cryostat sections from 5 equidistant rostrocaudal brain coronal sections, at −1.6, −0.8, 0, 0.8, and 1.6 mm from bregma, were selected. In each section, 5 randomly selected fields from the cortex ischemic core (420 μm × 420 μm) were analyzed and averaged per mouse, as we previously described (Bell et al., 2012; Nikolakopoulou et al., 2019).

### Extravascular Leakages

Perivascular capillary blood-derived IgG and fibrinogen deposits indicating BBB breakdown were measured as we previously described (Nikolakopoulou et al., 2019). The IgG-positive and the fibrinogen-positive perivascular capillary deposits on the abluminal side of CD31-positive endothelial profiles on microvessels <6 μm in diameter in the ischemic and non-ischemic cortex were analyzed using the ImageJ. Five animals per group were analyzed.

### Pericyte Coverage

Pericyte coverage analysis was performed similar to previously described (Bell et al., 2012; Nikolakopoulou et al., 2017). In brief, CD13 (pericyte) and CD31 (endothelium) staining was performed in methanol fixed tissue sections and signals from microvessels ≤6 μm in diameter were separately subjected to threshold processing. The areas occupied by their respective signals were analyzed using the ImageJ Area measurement tool. Pericyte coverage was quantified as a percentage (%) of CD13-positive pericyte surface area covering CD31-positive capillary surface area. Five animals per group were analyzed.

### Microvascular Length

The length of CD31-positive capillary profiles was determined similar to previously reported (Bell et al., 2012; Nikolakopoulou et al., 2017). The capillary profile length (vessels ≤6 m in diameter) was measured using the ImageJ plug-in length analysis tool. The length was expressed in mm of CD31-positive vascular profiles per mm^3^ of brain tissue. Five animals per group were analyzed.

### Neurodegeneration

Fluoro-Jade C was used to determine the tMCAo-induced neurodegeneration in mice as described (Chen et al., 2009; Wang et al., 2013; Nikolakopoulou et al., 2019). Cellular injury of ischemic brain sections was imaged at low power using a BZ 9000 Fluorescence Microscope (Keyence, Osaka, Japan), similar to as we reported previously (Wang et al., 2013). The number of fluoro-Jade C-positive neurons was quantified using ImageJ. Briefly, a plugin was used to render uniform the fluorescence in all sections. Using a nucleus counter particle analysis plugin, the number of stained neurons from each brain section was automatically counted. Five animals per group were analyzed.

### Statistical Analysis

The sample size chosen for our animal experiments in this study was estimated based on our prior experience performing similar experiments (Wang et al., 2009, 2012, 2013). Data are presented as mean ± standard error of the mean (SEM). One-way analysis of variance followed by Tukey’s multiple comparisons test was used to determine statistically significant differences. *P* < 0.05 was considered statistically significant.

## Results

**FIGURE 1:**
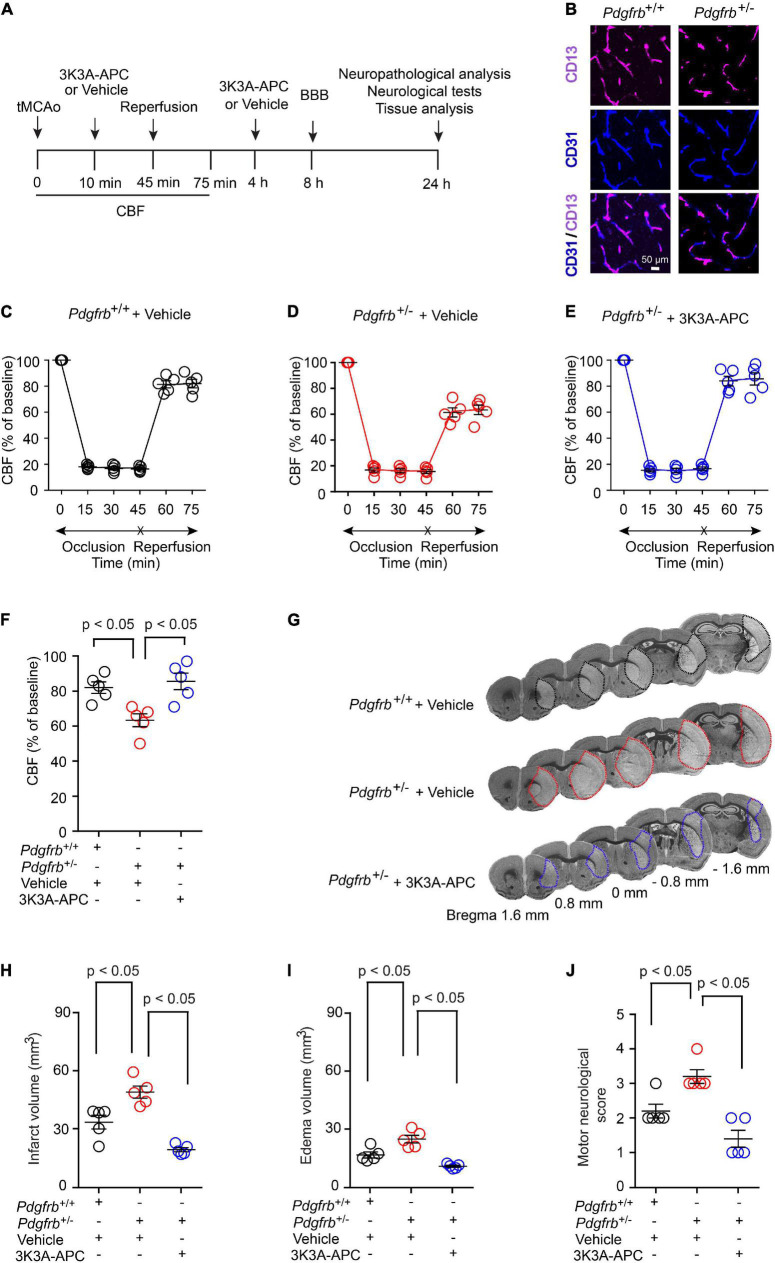
Accelerated ischemic brain injury in pericyte-deficient *Pdgfrb*^ + ⁣/−^ mice and protection by 3K3A-APC. **(A)** Scheme of the experimental set-up. Mice underwent 45-min transient middle cerebral artery occlusion (tMCAo) followed by reperfusion. 3K3A-APC (0.2 mg/kg i.v.) or vehicle were administered 10 min and 4 h after tMCAo. Cerebral blood flow (CBF) was monitored for the first 75 min. Blood-brain barrier (BBB) breakdown was evaluated by tissue analysis 8 h after tMCAo. Motor neurological score, neuropathological, and other tissue analyses (i.e., pericyte coverage, capillary length, neurodegeneration) were performed 24 h after tMCAo. **(B)** Immunostaining for endothelial (CD31) and pericyte (CD13) markers in non-ischemic cortex of *Pdgfrb*^+/+^ control mice and *Pdgfrb*^ + ⁣/−^ pericyte-deficient mice. **(C–F)** CBF determined by laser Doppler flowmetry during first 75 min of tMCAo in *Pdgfrb*^+/+^ controls **(C)** and *Pdgfrb*^ + ⁣/−^ mice treated with vehicle **(D)** or 3K3A-APC **(E)**. **(F)** CBF quantification at 75 min after tMCAo initiation in mice studied in **(C–E)**. **(G)** Representative images of cresyl violet staining of brain sections (at + 1.6, + 0.8, 0, –0.8, or –1.6 mm from the bregma) 24 h after tMCAo from *Pdgfrb*^+/+^ mice and *Pdgfrb*^ + ⁣/−^ mice treated with vehicle or 3K3A-APC. Infarct areas are outlined. Volumes of infarct **(H)** and edema **(I)**, and motor neurological score **(J)** 24 h after tMCAo in *Pdgfrb*^+/+^ mice and *Pdgfrb*^ + ⁣/−^ mice treated with vehicle or 3K3A-APC. In **(C–F,H–J)**, mean ± SEM, *n* = 5 mice per group. The *p*-values were calculated by one-way ANOVA followed by Tukey’s multiple comparisons test.

To investigate the effect of pericyte loss on ischemia, we induced stroke in 6-month-old *Pdgfrb*^ + ⁣/−^ mice and *Pdgfrb*^+/+^ littermate controls by 45 min tMCAo following the experimental scheme shown in Figure 1A. As previously reported (Bell et al., 2010; Kisler et al., 2017b), *Pdgfrb*^ + ⁣/−^ mice compared to *Pdgfrb*^+/+^ littermate controls develop a loss of brain capillary pericyte coverage within 6 months of age of approximately 27.5% as shown in Figure 1B. MCAo was confirmed by a stable >80% drop in CBF by LDF compared to basal pre-occlusion CBF values, as reported previously (Liu et al., 2003; Wang et al., 2012, 2013). In a treatment arm, *Pdgfrb*^ + ⁣/−^ mice received 3K3A-APC (0.2 mg/kg) or vehicle intravenously *via* tail vein 10 min and 4 h after tMCAo (Figure 1A). There were no significant differences in CBF reductions after MCAo between groups during the 45-min ischemia period (Figures 1C–E). However, significant differences between the groups were observed in the CBF recovery during an early period of reperfusion. At 75 min after tMCAo, CBF was restored to approximately 83% of baseline in control *Pdgfrb*^+/+^ mice that received vehicle (Figures 1C,F), but to only 62% of baseline in pericyte-deficient *Pdgfrb*^ + ⁣/−^ mice treated with vehicle (*P* < 0.01; Figures 1D,F), representing 26% greater loss of CBF during early reperfusion. Notably, treatment of *Pdgfrb*^ + ⁣/−^ mice with 3K3A-APC significantly improved CBF during the early reperfusion phase to 86% of the baseline compared to vehicle (*P* < 0.01) (Figures 1E,F), representing 38% improvement over vehicle-treated *Pdgfrb*^ + ⁣/−^ mice (Figure 1F).

*Pdgfrb*^ + ⁣/−^ mice treated with vehicle had increased cresyl violet-negative injury volume 24 h after tMCAo compared to control *Pdgfrb*^+/+^ mice that received vehicle (Figure 1G). This has been additionally confirmed by 43% and 49% increases in the infarct (Figure 1H) and edema (Figure 1I) volumes, respectively. 3K3A-APC treatment compared to vehicle significantly reduced the infarct and edema volumes in *Pdgfrb*^ + ⁣/−^ mice by 61 and 57%, respectively (Figures 1H,I). Similarly, vehicle-treated *Pdgfrb*^ + ⁣/−^ mice scored 50% worse on motor neurological score tests 24 h after tMCAo compared to control littermates (*P* < 0.05), but 3K3A-APC treatment significantly improved the motor neurological score in *Pdgfrb*^ + ⁣/−^ mice to a level similar to or better than in control littermates after tMCAo (Figure 1J). Together these data indicate that *Pdgfrb*^ + ⁣/−^ mice compared to *Pdgfrb*^+/+^ controls had impaired post-ischemic CBF reperfusion and are more prone to develop ischemic brain injury, likely due to loss of pericyte-mediated BBB protection (Montagne et al., 2018; Nikolakopoulou et al., 2019) and neurotrophic support (Nikolakopoulou et al., 2019). We also show substantial beneficial effects of 3K3A-APC treatment of stroked *Pdgfrb*^ + ⁣/−^ mice including significantly better early post-ischemic reperfusion and significantly improved neuropathological and functional outcome.

**FIGURE 2:**
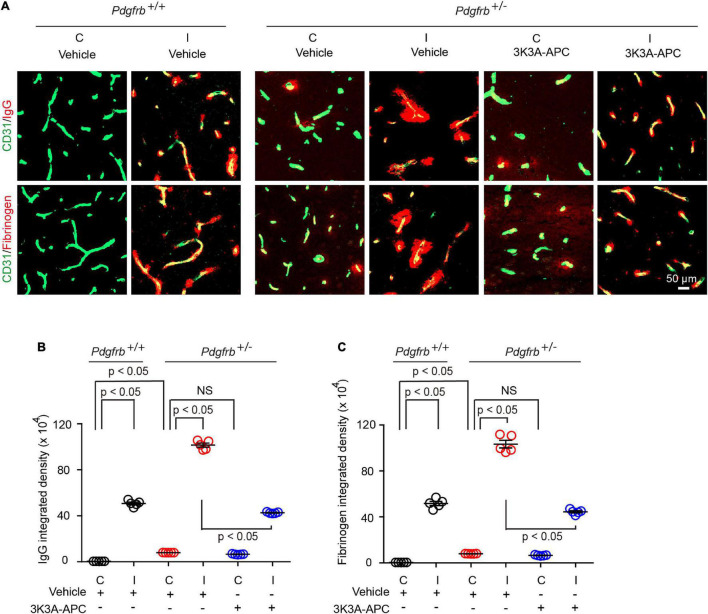
3K3A-APC protects the blood-brain barrier of pericyte-deficient *Pdgfrb*^ + ⁣/−^ mice from post-ischemic damage. **(A)** Immunostaining for endothelial marker CD31 and capillary (<6 μm in diameter) perivascular deposits of blood-derived immunoglobulin G (IgG, upper row) and fibrinogen (lower row) in non-ischemic contralateral cortex (“C”) and ischemic core of the ipsilateral cortex (“I”) 8 h after tMCAo in *Pdgfrb*^+/+^ mice and *Pdgfrb*^ + ⁣/−^ mice treated with vehicle or 3K3A-APC. Quantification of pericapillary (<6 μm in diameter) IgG **(B)** and fibrinogen **(C)** deposits in non-ischemic contralateral cortex (“C”) and ischemic core of the ipsilateral cortex (“I”) 8 h after tMCAo in *Pdgfrb*^+/+^ mice and *Pdgfrb*^ + ⁣/−^ mice treated with vehicle or 3K3A-APC. In **(B,C)**, mean ± SEM, *n* = 5 mice per group. The *p*-values were calculated by one-way ANOVA followed by Tukey’s multiple comparisons test.

Next, we evaluated the extent of BBB disruption and leakage of blood-derived products into the brain after tMCAo. In experimental models of transient focal cerebral ischemia, BBB disruption was observed at 6 h after reperfusion (Okada et al., 2020). In our previous study (Zhu et al., 2010), the most pronounced BBB leakage in wild-type untreated mice was observed within 8 h of reperfusion after tMCAo. Therefore, in the present tMCAo model we performed BBB measurements 8 h after reperfusion. Pericyte-deficient *Pdgfrb*^ + ⁣/−^ mice typically exhibit BBB leakage and extravasation of blood-derived molecules at an early age which progresses over time resulting in a significant BBB breakdown at 6 months of age (Bell et al., 2010). Here, we found that that post-ischemic BBB injury was much more pronounced in *Pdgfrb*^ + ⁣/−^ mice compared to *Pdgfrb*^+/+^ mice as shown by approximately 50% greater accumulation of pericapillary IgG and fibrinogen deposits in the ischemic core (Figures 2A–C). As previously reported (Bell et al., 2010), *Pdgfrb*^ + ⁣/−^ compared to *Pdgfrb*^+/+^ mice had by ∼10-fold higher accumulation of IgG and fibrinogen deposits in non-ischemic contralateral control hemisphere, but these deposits were an order of magnitude lower compared to deposits seen after ischemic injury in the ipsilateral stroked hemisphere (Figures 2B,C). BBB breakdown in *Pdgfrb*^ + ⁣/−^ mice has been shown to be associated with an age-dependent progressive loss of endothelial tight junction proteins such as zonula occludens-1 and occludin (Bell et al., 2010).

Treatment of *Pdgfrb*^ + ⁣/−^ mice with 3K3A-APC compared to vehicle significantly decreased extravascular IgG and fibrin accumulation by 58 and 57%, respectively (Figures 2A–C), indicating that 3K3A-APC aids in protecting BBB integrity. Although the present short-term treatment with 3K3A-APC did not improve broken BBB in the non-ischemic contralateral hemisphere in *Pdgfrb*^ + ⁣/−^ mice, it remains to be seen whether longer time therapeutic regimens with 3K3A-APC, as used, for example, in ALS (Zhong, 2009) and AD (Lazic et al., 2019) models, would alleviate BBB breakdown in *Pdgfrb*^ + ⁣/−^ mice that are not challenged by stroke.

**FIGURE 3:**
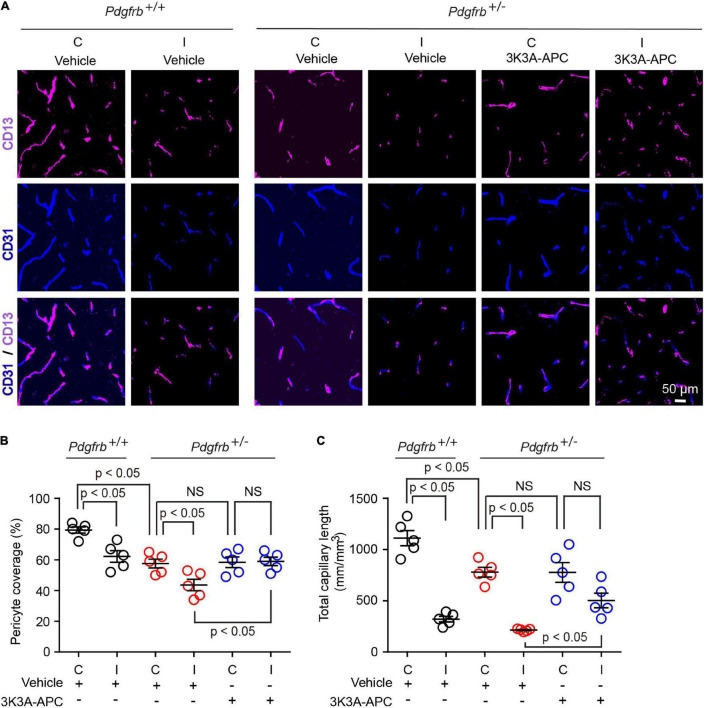
3K3A-APC alleviates post-ischemic loss of pericyte coverage and capillary microvascular length in pericyte-deficient *Pdgfrb*^ + ⁣/−^ mice. **(A)** Immunostaining for endothelial (CD31) and pericyte (CD13) markers in non-ischemic contralateral cortex (“C”) and ischemic core of the ipsilateral cortex (“I”) 24 h after tMCAo in *Pdgfrb*^+/+^ mice and *Pdgfrb*^ + ⁣/−^ mice treated with vehicle or 3K3A-APC. Quantification of CD13^+^-pericyte coverage of CD31^+^-endothelial capillary (<6 μm in diameter) profiles **(B)** and total length of CD31^+^-endothelial capillary profiles **(C)** in non-ischemic contralateral cortex (“C”) and ischemic core of the ipsilateral cortex (“I”) 24 h after tMCAo in *Pdgfrb*^+/+^ mice and *Pdgfrb*^ + ⁣/−^ mice treated with vehicle or 3K3A-APC. In **(B,C)**, mean ± SEM, *n* = 5 mice per group. The *p*-values were calculated by one-way ANOVA followed by Tukey’s multiple comparisons test.

Since a few studies suggested pericyte loss in models of large artery infarcts (Fernández-Klett et al., 2013; Nirwane et al., 2019; Ding et al., 2020; Zhang et al., 2020; Tsao et al., 2021) and bilateral carotid occlusion (Liu et al., 2019), we next evaluated the effect of tMCAo on the pericytes coverage in the present models. At 24 h after tMCAo, we found significant 23% loss of pericyte coverage in the ischemic core of *Pdgfrb*^+/+^ mice compared to the non-ischemic contralateral hemisphere, which was further accelerated by pericyte loss in *Pdgfrb*^ + ⁣/−^ mice treated with vehicle resulting in an absolute drop in pericyte coverage to 40% (Figures 3A,B). Treatment of *Pdgfrb*^ + ⁣/−^ mice with 3K3A-APC compared to vehicle almost completely prevented post-ischemic loss of pericytes (Figures 3A,B). Consistent with ischemic injury to microvasculature and loss of vessels (Fernández-Klett et al., 2013; Tsao et al., 2021), we also observed a substantial 75% loss of capillaries in the ischemic core in control *Pdgfrb*^+/+^ mice, which was similar to that found in *Pdgfrb*^ + ⁣/−^ mice treated with vehicle (Figures 3A,C). Treatment of *Pdgfrb*^ + ⁣/−^ mice with 3K3A-APC compared to vehicle ameliorated loss of capillaries by 50% (Figure 3C) consistent with its vasculoprotective activity (Griffin et al., 2015, 2018; Amar et al., 2018).

**FIGURE 4:**
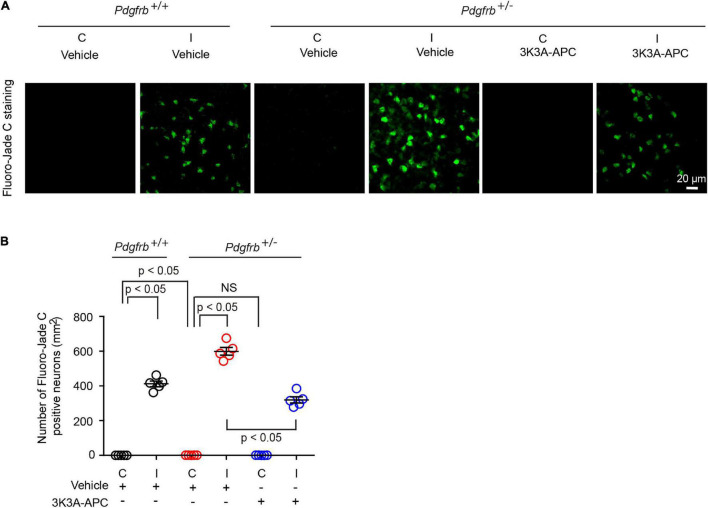
3K3A-APC reduces post-ischemic neurodegeneration in pericyte-deficient *Pdgfrb*^ + ⁣/−^ mice. **(A)** Fluoro-Jade C staining in non-ischemic contralateral cortex (“C”) and ischemic core of the ipsilateral cortex (“I”) 24 h after tMCAo in *Pdgfrb*^+/+^ mice and *Pdgfrb*^ + ⁣/−^ mice treated with vehicle or 3K3A-APC. **(B)** Quantification of Fluoro-Jade C^+^ neurons in non-ischemic contralateral cortex (“C”) and ischemic core of the ipsilateral cortex (“I”) 24 h after tMCAo in *Pdgfrb*^+/+^ mice and *Pdgfrb*^ + ⁣/−^ mice treated with vehicle or 3K3A-APC. Mean ± SEM, *n* = 5 mice per group. The *p*-values were calculated by one-way ANOVA followed by Tukey’s multiple comparisons test.

Finally, we studied the effects on neurons using Fluoro-Jade C assay (Chen et al., 2009; Wang et al., 2013; Nikolakopoulou et al., 2019). We observed Fluoro-Jade C-positive neurons in the ischemic hemisphere in all conditions 24 h after tMCAo (Figure 4A), indicative of neurodegeneration, whereas no Fluoro-Jade C-positive neurons were detectable in the contralateral non-ischemic hemisphere. After tMCAo, *Pdgfrb*^ + ⁣/−^ mice showed 35% more Fluoro-Jade C-positive neurons compared to control littermates (Figure 4B). Here again, 3K3A-APC treatment of *Pdgfrb*^ + ⁣/−^ mice compared to vehicle significantly decreased the number of Fluoro-Jade C-positive neurons by 47% (Figures 4A,B) consistent with its direct neuronal protective activity (Guo et al., 2009a,b; Griffin et al., 2015, 2018; Amar et al., 2018).

## Discussion

Our data show that pericyte-deficient *Pdgfrb*^ + ⁣/−^ mice (Bell et al., 2010; Kisler et al., 2017b) challenged by 45-min tMCAo develop accelerated post-ischemic brain injury compared to control *Pdgfrb*^+/+^ littermates. This has been shown by neuropathological analysis and functional tests indicating approximately 45–50% increases in the infarct and edema volume and motor neurological score 24 h after tMCAo. We also found that stroked *Pdgfrb*^ + ⁣/−^ mice have a 26% loss of CBF recovery during early reperfusion phase, a substantial BBB breakdown as shown by approximately 50% increases in perivascular capillary IgG and fibrinogen deposits 8 h after tMCAo, as well as 35% and 55% greater losses of pericyte coverage and the number of degenerating neurons as shown 24 h after tMCAo, respectively. We next show that 3K3A-APC, a signaling-selective analog of APC with greatly reduced anticoagulant activity (Mosnier et al., 2004, 2007, 2014; Guo et al., 2013), when administered intravenously to *Pdgfrb*^ + ⁣/−^ mice 10 min and 4 h after tMCAo, normalized CBF during the early reperfusion phase and reduced infarct and edema volume and motor neurological score by 55–60%, with similar reductions in post-ischemic BBB breakdown and the number of degenerating neurons.

In this first study in stroked pericyte-deficient mice, we focused on investigating the effect of pericyte loss and protection by 3K3A-APC using an acute stroke model. Future studies measuring the infarct volume, neurological deficits, survival rates, and underlying mechanisms at longer time points such as 3–7 days after 2–3 h tMCAo or permanent MCAo are needed to evaluate further the effects of pericyte deficiency on the pathophysiology of subacute stroke and the therapeutic effect of 3K3A-APC on later time points outcome in pericyte-deficient mice. However, these studies are beyond the scope of the present article concentrated on acute stroke.

Previous studies have shown that ischemic stroke in humans and mice leads to loss of brain pericytes associated with BBB disruption (Nirwane et al., 2019; Ding et al., 2020; Zhang et al., 2020; Tsao et al., 2021). Consistent with the high susceptibility of pericytes to hypoxic and ischemic injury (Peppiatt et al., 2006; Yemisci et al., 2009; Hall et al., 2014; Montagne et al., 2018), the present study confirmed post-ischemic pericyte loss in control *Pdgfrb*^+/+^ mice, and extended these observations by showing that pericyte deficiency in *Pdgfrb*^ + ⁣/−^ mice (Bell et al., 2010; Kisler et al., 2017b) results in even more severe post-ischemic pericyte injury and loss likely contributing to substantially larger post-ischemic BBB leaks. In addition to ischemia, it is also possible that considerably greater fluxes of blood-derived toxic fibrinogen in *Pdgfrb*^ + ⁣/−^ compared to *Pdgfrb*^+/+^ mice may present an extra challenge on already reduced pericyte capability to address the elevated fibrinogen burden, which has been shown to lead to autophagy-mediated pericyte cell death in another pericyte-deficient *Pdgfrb^F7/F7^* mouse line with 7 point mutations in the *Pdgfrb* gene causing impaired PDGFRβ signaling and in cultured mouse pericytes when challenged with fibrinogen *in vitro* (Montagne et al., 2018). We also confirmed a sizeable loss of capillary blood vessels after stroke in both *Pdgfrb*^ + ⁣/−^ and *Pdgfrb*^+/+^ mice consistent with previously shown losses of brain capillaries in wild-type stroked mice (Fernández-Klett et al., 2013; Tsao et al., 2021).

We also observed a much greater number of post-ischemic degenerating neurons in *Pdgfrb*^ + ⁣/−^ compared to *Pdgfrb*^+/+^ mice. It is possible that both greater BBB breakdown to different blood-derived neurotoxic products such as thrombin, plasminogen, red-blood cell-derived hemoglobin and toxic iron species, and/or others (Zhao et al., 2015) might act synergistically on ischemic neurons to amplify the effects of post-ischemic injury. It is also possible that a greater loss of pericyte-secreted neurotrophic factors, such as pleiotrophin, caused by greater post-ischemic pericyte loss, may lessen the overall protection of neurons from ischemic stress, as previously shown in pericyte-deficient *Pdgfrb^F7/F7^* mice subjected to ischemic and/or excitotoxic stress (Nikolakopoulou et al., 2019). However, the precise molecular mechanism(s) mediating neuronal injury in stroked *Pdgfrb*^ + ⁣/−^ mice remains to be determined by future studies that are beyond the scope of the present study.

Given that ischemia-induced pericyte contraction can contribute to the no-reflow phenomenon (Yemisci et al., 2009), one might expect that reduced pericyte numbers would alleviate no-reflow due to ischemia-induced pericyte contraction and result in a better outcome after stroke, which contrasts with what we observed. Interestingly, several studies have shown that manipulations that resulted in better retention or increased vascular pericyte coverage lessened post-ischemic brain pathology (Özen et al., 2018; Roth et al., 2019; Zhang et al., 2020) and also that inhibition of pericyte contraction improves cerebral microcirculation (Guo et al., 2021). Since it is estimated that a majority of CBF resistance is due to blood flow through the capillary bed (Gould et al., 2017), differences in capillary pericyte coverage and function may have an unexpectedly large effect on CBF reflow and recovery after reperfusion. On the other hand, it is possible that a greater degree of post-ischemic pericyte injury in *Pdgfrb*^ + ⁣/−^ compared to *Pdgfrb*^+/+^ mice might lead to a greater degree of ischemia-induced pericyte contraction (Yemisci et al., 2009; Hall et al., 2014) or dysregulation in remaining pericytes, which could contribute at least in part to deficits in post-ischemic CBF reperfusion as we observed. As 3K3A-APC has been reported to protect endothelial cells and neurons after ischemia (Griffin et al., 2015, 2018; Amar et al., 2018), it is possible that 3K3A-APC acts similarly to preserve pericytes and pericyte coverage, and reduce ischemia-induced pericyte contraction.

Finally, we showed that treatment of *Pdgfrb*^ + ⁣/−^ mice with 3K3A-APC protects the brain from accelerated ischemic injury caused by pericyte deficiency. The exact molecular and cellular mechanisms underlying BBB stabilizing and neuronal protective effects of 3K3A-APC in pericyte-deficient mice remain to be determined by future studies. 3K3A-APC is a multiple-target multiple action agent (Griffin et al., 2015, 2018; Amar et al., 2018). Its BBB-protection could be mediated by direct endothelial protective effects including stabilization of cytoskeleton *via* activation of Rac Family Small GTPase 1 (Rac1), suppression of nuclear factor-κB-mediated expression of the BBB-degrading matrix-metalloproteinase-9 (MMP9) enzyme, and suppression of inflammatory cytokines, as well as by inhibition of the intrinsic and extrinsic caspase-8 and caspase-9 pro-apoptotic pathways in ischemic endothelium, respectively, as shown by multiple independent studies as recently reviewed (Griffin et al., 2015, 2018; Amar et al., 2018). Endothelial-protective effects of APC and 3K3A-APC require activation of the protease-activated receptor-1 (PAR1), the presence of endothelial protein C receptor (EPCR), and/or co-activation of sphingosine 1 phosphate receptor 1 (S1P1), as documented by several laboratories and recently reviewed (Griffin et al., 2015, 2018; Amar et al., 2018). Here, we also showed that 3K3A-APC increased capillary coverage by pericytes, which may further contribute to stabilization of the BBB, since pericytes critically maintain BBB integrity (Armulik et al., 2010; Bell et al., 2010; Daneman et al., 2010; Nikolakopoulou et al., 2019). The exact mechanism of pericyte protection by 3K3A-APC is not clear at present, but likely involves direct protection from ischemic injury since pericytes are highly susceptible to hypoxic and ischemic injury (Peppiatt et al., 2006; Yemisci et al., 2009; Hall et al., 2014; Montagne et al., 2018). 3K3A-APC also exerts direct neuronal protective activity by blocking extrinsic and intrinsic pro-apoptotic pathways *via* PAR1 and PAR3 (Griffin et al., 2015, 2018; Amar et al., 2018), and protects oligodendrocytes from ischemic injury and inhibits post-ischemic pro-inflammatory astrocytes and microglia response, which also requires activation of PAR1 and PAR3, as recently shown (Huuskonen et al., 2022).

The present findings in pericyte-deficient mice are consistent with 3K3A-APC’s direct vasculoprotective and neuronal protective effects (Griffin et al., 2015, 2018; Amar et al., 2018) as previously shown in rodent models of stroke (Guo et al., 2009a; Wang et al., 2012, 2013; Sinha et al., 2018; Huuskonen et al., 2022), traumatic brain injury (Walker et al., 2010), ALS (Zhong, 2009; Shi et al., 2019), and AD (Lazic et al., 2019). These previous findings led to development of 3K3A-APC as a neuroprotectant in ischemic stroke (Williams et al., 2012; Lyden et al., 2013), with a safe, well-tolerated, and anti-hemorrhagic profile as shown in a Phase 2 clinical trial (Lyden et al., 2019). Since pericyte deficiency is associated with normal aging, and since accelerated loss of pericytes is found in neurodegenerative disorders such as AD (Sengillo et al., 2013; Halliday et al., 2016; Ding et al., 2020), ALS (Winkler et al., 2013), and others as reviewed elsewhere (Sweeney et al., 2019), the present findings may have implications for treatment of ischemic brain injury in different neurological conditions associated with pericyte loss.

## Data Availability Statement

The raw data supporting the conclusions of this article will be made available by the authors, without undue reservation.

## Ethics Statement

The animal study was reviewed and approved by the Institutional Animal Care and Use Committee at the University of Southern California.

## Author Contributions

YW, KK, and BZ contributed to the experimental design. YW conducted experiments, analyzed the data, and prepared the figures. AN and KK contributed to data analysis and data presentation. JF and JG provided reagents. KK and YW contributed to manuscript writing. BZ supervised all data analysis and interpretation and wrote the manuscript. All authors contributed to the article and approved the submitted version.

## Conflict of Interest

JG reports the patent US 7,498,305 that is licensed to ZZ Biotech LLC and he is a consultant for ZZ Biotech LLC. BZ was a founder of ZZ Biotech LLC, a biotechnology company with a mission to develop APC and its functional mutants for the treatment of stroke and other neurological disorders. The remaining authors declare that the research was conducted in the absence of any commercial or financial relationships that could be construed as a potential conflict of interest.

## Publisher’s Note

All claims expressed in this article are solely those of the authors and do not necessarily represent those of their affiliated organizations, or those of the publisher, the editors and the reviewers. Any product that may be evaluated in this article, or claim that may be made by its manufacturer, is not guaranteed or endorsed by the publisher.
